# A Mini-Review on Multi-Hurdle Control of *Salmonella* Along Poultry Production Continuum

**DOI:** 10.3390/ani15060875

**Published:** 2025-03-19

**Authors:** Eniola Betiku, T. Tiwa Ogundipe, Tanmaie Kalapala, Tomi Obe

**Affiliations:** 1 Department of Poultry Science, University of Arkansas System Division of Agriculture, Fayetteville, AR 72701, USA; ebetiku@uark.edu (E.B.); tanmaiek@uark.edu (T.K.); 2 Department of Food Science, Center for Food Safety, University of Arkansas System Division of Agriculture, Fayetteville, AR 72704, USA

**Keywords:** *Salmonella*, poultry production, pre-harvest strategies, post-harvest interventions, food safety, multi-hurdle control

## Abstract

This mini-review presents common strategies for controlling *Salmonella* in poultry, addressing combined pre-harvest and post-harvest interventions to create a multi-hurdle approach. The goal is to highlight integrated approaches that enhance overall food safety and sustainability within the poultry industry. Current pre-harvest and post-harvest strategies are discussed, including industry practices and regulatory frameworks. Emphasis is placed on the implementation of biosecurity measures, vaccination, feed management, and environmental control in pre-harvest settings, as well as processing plant interventions such as antimicrobials for carcass decontamination, sanitation, and quality control measures. Pre-harvest strategies that have shown promise include enhanced biosecurity protocols, selective vaccinations for pathogenic *Salmonella* strains, and advanced feeding regimens. Post-harvest interventions, such as antimicrobial application for decontamination, have improved sanitation practices, and pathogen reduction technologies are also critical in reducing *Salmonella* prevalence. An integrated approach that combines both pre-harvest and post-harvest measures is essential for an effective *Salmonella* control program. Implementing a continuum of control strategies for *Salmonella* in poultry production is vital for ensuring food safety and protecting public health. Collaborative efforts between researchers, industry stakeholders, and policymakers are necessary to address emerging issues and enhance overall effectiveness.

## Simple Summary

*Salmonella* contamination in poultry remains a significant public health concern, impacting both the poultry industry and consumers. Effective control measures must span the entire poultry production continuum, from farm to table, to mitigate the risk of contamination and outbreaks.

## 1. Introduction

Poultry has emerged as the most consumed meat product, and it is projected to remain the world’s largest imported livestock commodity by volume over the next decade [1,2]. In 2023, broiler chicken was the most consumed animal protein in the United States, with per capita consumption reaching approximately 99.5 pounds [3]. This consumption rate is expected to increase to around 105 pounds in 2025, reflecting the ongoing consumer preference for poultry products [3]. This surge in demand can be attributed to affordability, nutritional value, and versatility in culinary applications [1,4]. As a result, domestic production has significantly increased and continued to increase in the United States [1,5], which stands as the world’s largest producer and second largest exporter of broilers [5]. Poultry production involves multiple stages before products reach consumers. For broilers, it begins with raising chicks from hatch to performance, followed by processing, resulting in diverse poultry products for consumption or use as by-products [6]. In the United States, the poultry industry operates through a vertically integrated system encompassing production (hatcheries, feed mills, breeders, and broilers), processing (deboning, cutting, and packaging), and distribution [7]. This system enhances efficiency and quality control throughout the supply chain. Furthermore, U.S. poultry production comprises both commercial and small-scale operations, which differ in production and management approaches. The commercial sector, which includes broiler chickens, turkeys, and table egg layers, relies on controlled breeding programs to manage genetics and optimize productivity [8]. Here, broiler chickens and turkeys are transferred directly from hatcheries to broiler or turkey grower farms [9]. Small-scale poultry operations, including backyard poultry and specialty poultry like pasture, free-range, cage-free, and organic operations, sometimes involve multiple sources and may have contact with each other, not adhering to the all-in-all-out management systems typically seen in commercial operations [9,10,11]. This variability can introduce challenges in disease management and biosecurity [10]. In fact, poultry has been identified as a significant reservoir for foodborne pathogens, notably *Salmonella* and *Campylobacter* [7]. Consequently, food safety is a critical consideration across all stages of the poultry production continuum from pre-harvest (farm) to post-harvest (processing plants) and consumers. While many mitigation efforts have been developed, evaluated, and found to be effective, these enteric pathogens remain a significant food safety and public health challenge [12]. Therefore, continued efforts to enhance safety standards and educate consumers are essential to protect public health while meeting the growing demand for poultry products. This mini-review combines common strategies that have been reported and proven at pre-harvest and post-harvest production stages and discussed strategies to integrate these systems to create more comprehensive multi-hurdle and multi-technology approaches, as combined efforts are better than a single strategy.

## 2. *Salmonella* Complexity in Poultry Production

*Salmonella enterica* is one of the most significant pathogens in poultry production, posing a significant risk to food safety and public health [13]. This pathogen is diverse, having many serotypes that are categorized into two primary illness types. Typhoidal *Salmonella* causes illnesses like typhoid fever, a severe illness that can be life-threatening if untreated, and non-typhoidal *Salmonella* is commonly linked to foodborne infections that can spread through contaminated poultry products [14]. Nontyphoidal *Salmonella* infection can cause symptoms ranging from mild to severe gastrointestinal issues such as abdominal pain, diarrhea, fever, and nausea with potential long-term health problems like reactive arthritis [15,16]. According to the Centers for Disease Control and Prevention (CDC), *Salmonella* infections are usually self-limiting, lasting 4–7 days, but can last longer when the infection spreads to other parts of the body, leading to complications [16]. This diversity in *Salmonella enterica* is further expressed through host adaptation, environmental prevalence and persistence, and virulence. While some serotypes are broad host-adapted like the serotypes of Enteritidis and Typhimurium (non-typhoidal), others like Typhi (typhoidal) thrive in specific hosts [12,13,14]. *Salmonella enterica* includes over 2500 serotypes, and only a few, such as Enteritidis, Heidelberg, Infantis, I 4,[5],12:i:−, Kentucky, and Typhimurium, have been historically linked to poultry [17,18]. Interestingly, despite the prevalence of the Kentucky serotype in poultry, it is less frequently associated with salmonellosis, unlike others, e.g., Enteritidis, Infantis, I 4,[5],12:i:−, Newport, and Typhimurium [19,20,21,22]. Moreover, recent data indicate that the Infantis serotype has become an emergent serotype in post-harvest poultry production in the United States [20]. These serotypes are unique and diverse in colonization and virulence in poultry and humans [13,20] and differ in their response to common mitigation strategies.

Nontyphoidal *Salmonella* colonizes the intestinal tract of poultry, causing the contamination of meat during processing and handling. Poultry and its products are vulnerable to contamination at various points in the production chain, from farms to processing plants. On farms, *Salmonella* can be introduced through multiple sources, such as contaminated feed and the farm environment through water, equipment, and personnel [13,23,24]. Vertical transmission from breeder flocks to chicks is a critical route, by which infected hens can transmit *Salmonella* directly to their offspring via contaminated eggs [25]. Horizontal transmission is also common, occurring among birds through direct contact and shared spaces [13]. At the processing stage, cross-contamination can occur during slaughtering, defeathering, evisceration, and sectioning [13]. Improper handling of carcasses during scalding, poor hygiene practices in evisceration, poor cleaning and sanitation of surfaces and equipment, or inadequate antimicrobial treatment can further facilitate the spread of *Salmonella*, increasing the risk of contaminated products reaching consumers [20,26]. The effective control of *Salmonella* will require a comprehensive strategy throughout the production continuum, from farms to processing plants.

With diverse serotypes exhibiting varying levels of pathogenicity [20,27], managing *Salmonella* requires a comprehensive, risk-based approach across the entire food production chain. Key strategies for controlling *Salmonella* at the farm level include the use of synthetic and natural compounds, such as vaccines, to enhance the immune response in chickens [13,25]. Effective litter management, feed fortification with probiotics and/or prebiotics, and drinking water sanitation are common methods employed to reduce the pathogen load [28,29]. These measures aim to limit *Salmonella* colonization and spread among poultry populations. In poultry processing plants, multi-hurdle technology is applied to further reduce *Salmonella* levels. This includes the use of various chilling methods, chemical treatments, and mechanical interventions designed to minimize bacterial contamination [30,31,32,33]. For example, chilling conditions can be optimized to inhibit bacterial growth, while chemical washes or sprays may be used to kill bacteria on carcass surfaces [31]. Further, *Salmonella* control during processing relies on effective monitoring programs, adherence to good manufacturing practices (GMPs), and Hazard Analysis Critical Control Point (HACCP) protocols [34]. These programs ensure that contamination is detected and addressed promptly, maintaining the safety of poultry products for consumers.

**Figure 1:**
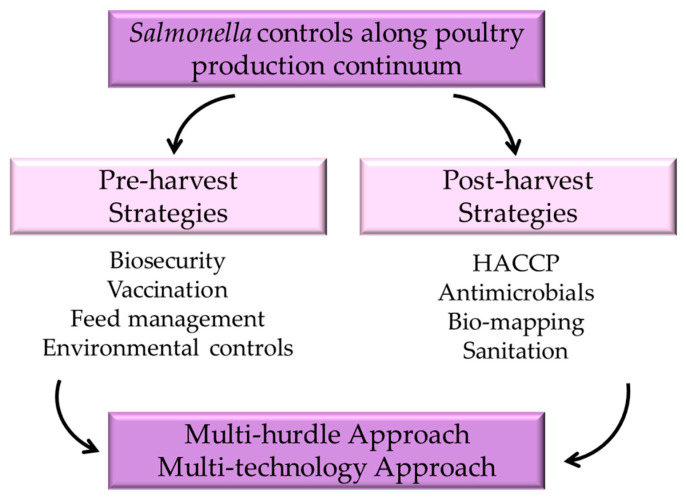
Strategies to control *Salmonella* in poultry production.

Given the complexity of *Salmonella*, increased knowledge about specific serotypes through surveillance and monitoring will enable more targeted interventions [20]. Different serotypes may respond differently to control measures, so tailored approaches can enhance the effectiveness of interventions [35]. Ultimately, controlling *Salmonella* within the poultry industry is a collaborative effort involving producers, processors, and consumers. Each stakeholder plays a crucial role in ensuring that poultry products are safe for consumption. Producers must implement effective on-farm practices, processors need to apply rigorous interventions at the plant level, and consumers need to practice safe food handling to mitigate the risk of *Salmonella* infection (Figure 1). To address these challenges, the industry is increasingly adopting advanced technologies and practices, such as improved biosecurity measures and enhanced surveillance systems that evaluate serotype populations [12,18,20,36], as well as the use of novel feed additives and vaccine preparations like electron-beam-inactivated vaccines [37,38] to reduce pathogen loads. Additionally, ongoing research efforts aim to further improve poultry health and welfare while minimizing the environmental impact of production. More recently, whole genome sequencing (WGS), an application of genomics, has been used in the diagnosis, epidemiology, and surveillance of *Salmonella* [39,40].

## 3. Pre-Harvest Control Strategies

The USDA’s recently proposed framework, which could require the testing of incoming flocks for *Salmonella* before processing, has spurred renewed interest in understanding *Salmonella* dynamics within poultry production environments [41]. These interests include understanding the interactions between poultry and their environment and developing novel pre-harvest intervention strategies. As previously noted, poultry are particularly susceptible to *Salmonella* colonization from various sources during live production, including hatcheries, contaminated feed, breeder flocks, farm environments, litter, and during feed withdrawal [13,28,42]. Among these, the hatchery is one of the most significant contributors to *Salmonella* prevalence, with a reported 48.5% prevalence [28]. Consequently, obtaining *Salmonella*-free chicks is critical to reducing *Salmonella* incidence at this early stage. Intervention strategies targeting foodborne pathogens in pre-harvest broiler production focus on *Salmonella* and *Campylobacter*, specifically *Salmonella* serotypes frequently associated with human illness, such as Typhimurium and Enteritidis [7]. While *Salmonella* Kentucky is the most isolated in live production samples, it is less prevalent in processing plant samples, indicating that processing control measures are effectively mitigating this serotype [20,35,36].

*Salmonella* can contaminate raw feed ingredients at several stages of manufacturing. Factors influencing microbial multiplication at feed plants include moisture levels, feed composition, and thermal processing intensity [43]. The studies reviewed by [28] show that *Salmonella* prevalence in poultry feed can range from 0 to 100% and 0 to 40% according to studies within the United States. The removal of antibiotics from poultry production has created a demand for alternative feed amendments that can replicate some of the gut health and performance benefits traditionally provided by antibiotics [44,45]. Despite the need for alternatives, the effectiveness of some of these compounds as feed amendments has been mixed [46], especially since *Salmonella* is very diverse; hence, using a broad-spectrum antimicrobial agent that would be effective on multiple serotypes and strains is critical. In recent years, non-antibiotic alternatives in poultry production have gained attention [29]. These include feed-based interventions like probiotics, prebiotics, phytobiotics, and postbiotics [44,45,46]. The concept of competitive exclusion, in which beneficial bacteria outcompete pathogens for space and nutrients in the digestive tract, has been explored using probiotics [47,48,49,50]. Probiotics have shown potential in mitigating *Salmonella* infections [51], but many feed additives lack similar efficacy. More research is needed to identify effective feed additives with consistent efficacy. Beyond feed-based approaches, nonfeed-based alternatives, such as antimicrobials, vaccines, and in ovo strategies, are common interventions to combat the *Salmonella* burden in poultry [29,52]. Vaccination is a crucial health management strategy for boosting poultry flock immunity and is widely used in breeders and broilers to reduce *Salmonella* colonization and prevalence [29]. Vaccines are among the most effective and cost-efficient tools for preventing diseases in birds [53]. Typically, poultry vaccines use *Salmonella* serotypes Typhimurium and/or Enteritidis [54], which are broad-spectrum serotypes colonizing many food animals and humans and implicated in many foodborne outbreaks. Available vaccine types include live attenuated, inactivated, and subunit vaccines, offering various options for disease prevention [55,56]. Advances in *Salmonella* vaccines include the evaluation of electron-beam (eBeam) irradiation technology in vaccine preparation [57]. This has been used to prepare an inactivated *Salmonella* Enteritidis vaccine that was found to reduce cecal colonization [37]. Apart from single-serotype vaccines, this could potentially be used to evaluate multi-serotype vaccines for effective *Salmonella* control during pre-harvest poultry production.

In addition to enhancing flock performance through an effective feeding regimen and vaccination, effective poultry drinking water sanitation is vital for pre-harvest food safety, aiming to minimize foodborne pathogens and protect consumer health [58,59]. While the acceptable microbial load for poultry drinking water is 1000 CFUs per milliliter of aerobic bacterial count, the presence of *E*. *coli* and other pathogens is unacceptable (0 CFU/mL) [60]. *Salmonella* contamination in drinking water systems is typically minimal, with a prevalence ranging from 0 to 11% [28], and water treatment further reduces the risk. However, the optimal temperature (around 25 °C), low flow rates, and nutrient availability can promote microbial contamination and biofilm formation, complicating disinfection strategies [28,29,59]. Various chemical-based sanitizers are used to disinfect poultry drinking water, though their efficacy remains uncertain, highlighting the need to demonstrate the effectiveness of various water sanitation strategies. Biofilm removal in the drinking water system is also vital for the effective reduction in opportunistic and pathogenic microbial populations in the drinking water system [58,61]. Robust biosecurity measures are crucial in controlling *Salmonella* transmission and improving food safety [62]. Physical barriers, including fences, mesh wire, footbaths, and farm equipment disinfection, are key components of biosecurity programs [63,64]. Additionally, rodent and fly control, red mite management, and disinfection between flocks are recommended to reduce *Salmonella* incidence and disrupt disease cycles [65]. Proper litter management, such as composting, is essential in lowering *Salmonella* contamination, as fresh wood shavings are linked to higher contamination than older litter [66,67]. Biosecurity practices are among the most cost-efficient and effective preventive measures for managing disease risks to the economy, environment, and public health [68]. They not only reduce infectious disease risks but also hold regulatory importance. The U.S. Food Safety Modernization Act’s Preventive Control for Animal Food regulation is a valuable resource for ensuring poultry product safety [69]. In summary, an integrated approach encompassing control from the hatchery to the farm through feed amendments, vaccination, water sanitation, biosecurity measures, and regulatory compliance is essential for effectively controlling *Salmonella* in poultry production and ensuring pre-harvest food safety.

## 4. Post-Harvest Strategies

While pre-harvest strategies focus on preventing and managing contamination at the farm level, post-harvest interventions aim to minimize or eliminate contamination during the stages of processing, packaging, and distribution. Although the microbiological quality of poultry meat is influenced by the health of live birds, it is crucial to ensure there is no cross-contamination during processing operations. Government regulations are essential in establishing and enforcing standards for controlling *Salmonella* in the poultry industry [70]. The U.S. Department of Agriculture Food Safety and Inspection Service (USDA-FSIS) has set specific performance standards for *Salmonella* in poultry products [70]. These standards dictate a certain percentage of chicken samples that may test positive for *Salmonella*, ensuring food safety [30,70]. To comply with these standards, poultry processing plants are required to implement a Hazard Analysis Critical Control Point (HACCP) plan in addition to existing regulations with strict adherence to good manufacturing practices and thorough sanitation procedures throughout the processing stages [13,71]. These systems are designed to identify and manage potential hazards throughout the processing steps, and adherence to these guidelines can result in meaningful improvements in food safety [24]. While these guidelines provide a foundation, they have not always produced the desired food safety result. Poultry integrators have adopted additional strategies, including the bio-mapping of processing environments [72,73]. Microbial mapping, also called bio-mapping, has become an invaluable tool for monitoring and managing *Salmonella* contamination, allowing processors to identify contamination hotspots within the processing environment and promptly mitigate contamination risks [72,73,74,75]. This approach systematically samples and analyzes different areas within a processing plant to identify the locations most vulnerable to *Salmonella* contamination [76]. For example, ref. [74] evaluated the presence of *Salmonella* on chicken samples obtained after the bleeding, scalding, defeathering, carcass opening, evisceration, bird washing, prechilling, and chilling steps of poultry processing to demonstrate the utility of bio-mapping. This study revealed higher contamination levels, particularly after bleeding, scalding, defeathering, and evisceration [74]. Interestingly, there was also a decrease in contamination in areas to which specific treatments were applied. Likewise, ref. [75] evaluated *Salmonella* incidence across similar processing steps, with scalding and evisceration lines consistently being identified as high-risk areas. Furthermore, ref. [72] conducted a comprehensive bio-mapping study assessing not just the quantity of pathogens (*Salmonella* and *Campylobacter*) but also indicator organisms like aerobic bacteria and Enterobacteriaceae. This was performed under two different processing conditions, a normal chemical process with typical chemical interventions; and a reduced chemical process with minimal or no chemical interventions at different locations within a processing line, ranging from live receiving to post-chilling and parts (wings) processing [72]. The results suggest that the normal chemical process generally resulted in significantly lower *Salmonella* counts compared to the reduced chemical process at most locations. Generally, the study corroborates other studies [74,75,77] that highlight scalding, picking, and evisceration as key areas to reduce the bacterial load. These studies highlight the need for the effective disinfection of chicken carcasses at the primary processing (slaughter to chilling) stage to reduce the microbial load during secondary processing, including during deboning, portioning, and packaging [77].

Maintaining hygienic standards in poultry processing facilities is essential in reducing the risk of *Salmonella* contamination. Effective cleaning and sanitation, which include the routine application of approved cleaning agents and sanitizers, play a key role in this process [77]. This process is designed to minimize the presence of *Salmonella* on surfaces and equipment that are in direct contact with products [77]. Aside from equipment, poultry workers should maintain adequate hygienic standards. Workers are often in close contact during processing, making them potential vectors for cross-contamination if proper hygienic practices are not followed [78]. Commercial processing plants encourage frequent handwashing practices with the use of personal protective equipment (PPE) to prevent cross-contamination from employees. Commonly used sanitizers, such as chlorine-based compounds, peracetic acid (PAA), and quaternary ammonium compounds, have been recognized for their efficacy in processing plants [33,79,80]. To achieve comprehensive cleaning and sanitation, it is essential that processing equipment is hygienically designed to allow for easy access during cleaning and sanitation [77,81]. However, studies [77,82,83] have shown that *Salmonella* is resilient and continuously evolving past control strategies, persisting on processing surfaces after antimicrobial interventions. The authors [77] reported a high prevalence of *Salmonella* on several pieces of processing equipment, including the head puller, scalder, picker, and cropper, after sanitation. The prevalence varied between the plants evaluated in the study, and this could be due to variability in the sanitizers used by the plants and effective cleaning procedures. The application of sanitizers during processing stages such as scalding, eviscerating, internal/external bird washing, and chilling shows a paramount intervention strategy to ensure product safety; however, as previously noted, the use of sanitizers varies across processing facilities. Historically, chlorine has been widely used in poultry processing due to its cost effectiveness and the fact that it only requires a low concentration for pathogen reduction [33]. However, some studies have reported concerns about its actual effectiveness in certain applications within processing environments [32,84,85]. For instance, chlorine has been found to be less effective when applied as a spray [33,84] compared to other applications, like immersion. These authors suggest that spraying chlorine on carcass surfaces does not always reduce bacteria as expected, leading to bacterial persistence on carcasses and surfaces. Moreover, the use of higher concentrations of chlorine on poultry products has been associated with undesirable outcomes, such as the creation and release of trihalomethanes, a by-product of chlorine with organic compounds in water [86]. Peracetic acid (PAA), however, is the most popular disinfectant used in poultry processing [33]. Research has shown that even at low concentrations and permissible levels, PAA effectively reduces *Salmonella* post-chilling [31,84,87,88,89,90]. Ref. [31] compared PAA with chlorine and showed that PAA is more effective in reducing pathogen levels. Likewise, ref. [90] showed its efficacy was significantly better than cetylpyridinium chloride (CPC), achieving approximately 1.5 and 1.3 log_10_ reductions at 0.07 and 0.1% concentrations, respectively, while CPC achieved a 0.8 log reduction at 0.35 and 0.6%. Although the use of PAA in immersion methods typically shows more consistent outcomes, a review by [80] noted that spray applications tend to yield variable results. There is a growing interest in exploring the potential of combining PAA with other antimicrobials to enhance efficacy and prevent antimicrobial tolerance that could arise from consistent use. Quaternary ammonium compounds like CPC have been shown to be effective against *Salmonella* on poultry carcasses; however, the contact time set by the USDA could limit its usage, along with the need to perform a portable water rinse after its application [33,91,92]. In addition to these antimicrobials, other pathogen reduction technologies have been evaluated to control *Salmonella* in poultry products and during processing. Some of these approaches are electrochemically activated water (ECAW), ozone-based technologies, and bacteriophages [93,94,95]. However, some of these approaches have not been thoroughly evaluated, limiting our understanding of their efficacy. For instance, ECAW, which produces hypochlorous acid from the electrolysis of salt and water [93], was reported to reduce *Salmonella* counts in a simulated chiller environment at 200 ppm; however, this concentration is significantly higher than the 50 ppm recommended for contact with poultry carcasses in the chiller [91]. Likewise, ref. [94] evaluated ozonated water on chicken wings at three concentrations, 2.5, 5, and 10 ppm. The authors reported reduction levels lower than 1.0 log_10_ CFU/mL, which may not be practical for industry applications.

Despite the effectiveness of some of these interventions, *Salmonella* can persist on the surface of processing equipment and develop biofilms that are difficult to remove [82,96,97]. Factors like the improper usage of antimicrobials, exposure to sub-lethal concentrations, and temperature abuse could encourage biofilm formation and *Salmonella* persistence in processing environments [77,98,99]. Biofilms on processing equipment can protect *Salmonella* from sanitizers, allowing the survival and potential cross-contamination of subsequent batches of poultry products [77,83,100]. In addition, the quantification of *Salmonella* that persist on surfaces after processing is crucial for assessing cross-contamination risks, the effectiveness of sanitation practices, control measures, and public health risks. While current sanitary processing and processing measures have significantly reduced *Salmonella* contamination, there remains an ongoing need for continual improvements to address the challenges of emerging virulent serotypes, biofilm formation, antimicrobial tolerance and/or resistance, and other factors contributing to *Salmonella* survival and persistence.

## 5. Multi-Hurdle Approach to Controlling *Salmonella*

Combating *Salmonella* requires a multi-hurdle approach that aligns with a One Health perspective, emphasizing the interconnectedness of human, animal, and environmental health [101]. The complexity and adaptability of *Salmonella* necessitates a multifaceted approach to effectively lower contamination levels in poultry [102]. A summary of current strategies discussed in the review implemented individually or as a combined approach are highlighted in Table 1. However, no single intervention, whether it be pre-harvest measures or post-harvest strategies, has shown sufficient efficacy to significantly reduce *Salmonella* prevalence within the poultry production continuum [13,103,104]. This limitation highlights the challenges poultry integrators face in managing this pervasive pathogen that can persist under and adapt to diverse environmental conditions [77,96,105,106].

The multi-hurdle approach for controlling *Salmonella* in poultry is characterized by incorporating several interventions that work synergistically to minimize contamination risks [13,107,108,109,110,111]. This approach will integrate multiple control measures to create a composite effect that is more effective than individualized measures [112]. By deploying several strategies simultaneously, producers can target different stages of the poultry production chain, from pre-harvest to processing, thereby addressing potential contamination points (control points) more thoroughly. This comprehensive strategy will focus on identifying and addressing microbial hazards at various critical control points throughout the poultry production process and establishing mitigation plans. This plan will emphasize the importance of integrating controls from the earliest stages of poultry production, specifically during the pre-harvest phase, when management practices including biosecurity and *Salmonella*-free flocks at the breeder stage are critical [104,107]. This holistic methodology ensures that even if one control measure fails or is less effective, others will still contribute to overall pathogen reduction.

For instance, a successful pre-harvest multi-hurdle approach will employ combined interventions such as vaccination, a feeding strategy to induce competitive exclusion, water treatments for biofilm control and to improve gut health, and enhanced hygiene practices to prevent *Salmonella* and other enteric pathogens from proliferating in the birds [13,29,38,59]. A robust multi-hurdle vaccination program could include the established vaccination program in combination with the application of autogenous vaccines to maximize the *Salmonella* reduction. An autogenous vaccine is a custom vaccine prepared from *Salmonella* serotypes detected in a flock through surveillance and monitoring [113]. This vaccine is then used on subsequent flocks on the same farm. Post-harvest, the combination of strategies highlighted in Table 1 will ensure a continued reduction and prevent pathogen introduction into the supply chain. This method of using a combined approach will contribute to a more resilient food safety system by applying multiple corrective actions to tackle *Salmonella* contamination across the entire production chain [114]. While some studies have reported a significant reduction when two or more applications [109,115] are used, others have not seen similar effect [116], meaning there is a need for continued efforts to study the effectiveness of combining applications post-harvest. These efforts should also establish a methodology for applying certain measures, as combined synergistic applications differ from sequential synergistic applications [108,109,110,115].

It is noteworthy that the use of plant-derived, food-grade phytochemical nano emulsions represents a promising intervention in the multi-hurdle multi-technology approach to control *Salmonella* and other pathogens in poultry production [117,118,119]. These phytochemicals are developed from various plant sources and prepared in nano-emulsions to increase their dispersion in aqueous medium [120,121]. They are designed to be safe for consumption while effectively targeting pathogens. Authors have shown that employing such interventions can significantly reduce the colonization and presence of harmful bacteria in poultry, thereby enhancing overall food safety [120,121,122]. These efforts can be effectively combined with existing efforts like biosecurity measures restricting access to poultry farms, ensuring proper sanitation practices, managing external vectors that may carry pathogens, and other antimicrobial applications like bacteriophages [64,123,124].

Moreover, by optimizing factors like ventilation, litter management, and waste disposal, growers can create environments that are less conducive to the growth of harmful bacteria, significantly reducing contamination risks [125]. Reducing the microbial load on farms can significantly enhance the effectiveness of the processing strategies previously discussed. The United States Department of Agriculture (USDA) and the Food and Drug Administration (FDA) are key regulatory agencies that endorse the multi-hurdle approach in their guidelines for controlling *Salmonella* in poultry [104,126]. Their support reflects a recognition of the limitations of individual interventions and the need for a comprehensive strategy to manage food safety risks associated with poultry production. The regulatory emphasis on the multi-hurdle approach also encourages collaboration among stakeholders in the poultry industry, including producers, processors, and suppliers. Such coordination is vital for implementing effective interventions and aligning practices across the production continuum to ensure that each link contributes to the goal of reducing *Salmonella* contamination. This shared vision facilitates the development of best practices and innovative solutions, further enhancing food safety. The investigation of novel interventions like bacteriophages reflects a commitment to continually improving food safety protocols in poultry production [127,128]. Bacteriophages have continued to gain attention due to their ability to selectively infect and kill *Salmonella*, presenting a promising method for reducing its prevalence in poultry products without negatively impacting the broader microbiota essential for poultry health [95,129]. Their application could provide a complementary strategy within the multi-hurdle approach, potentially enhancing its overall effectiveness against *Salmonella*. By integrating cutting-edge technologies and biological solutions within the multi-hurdle approach, the industry aims to adapt to evolving challenges posed by pathogens like *Salmonella*, ensuring that poultry remains a safe choice for consumers.

**Table 1:** Management strategies and their mode of application in different studies.

Strategies	Type of Application	Application	References
Feed management	Prebiotics	In feed	[47,48,50,51]
	Probiotics, phytobiotics, postbiotics, feed additives	In feed	[28,29]
In ovo strategies	Bioactive substances	In Ovo	[52,130]
Vaccines	Live attenuated, inactivated, subunit, killed	In Ovo, oral, intramuscular	[29,37,38,54,55,57]
Bacteriophages	Lytic phage lysates	Intra-cloacal	[131]
	Encapsulated phage	In drinking water	[132,133]
	Lytic phage	Chicken breast fillet and skin	[115]
Drinking water management	Sanitizers	In drinking water	[58,59,60,61]
Biosecurity	Physical barriers	General on-farm practices	Physical barriers
	Rodent and fly control Red mite management	Sanitation protocols	[65,134]
Litter management	Fresh wood shavings	Composting	[66,67]
HACCP	Good manufacturing practices	Sanitation procedures	[24,30,34]
Bio-mapping	Identification of contamination hotspots	Sampling at processing	[72,73,74,75,77]
Antimicrobials	Peracetic acid (PAA)	In water processing	[31,32,84,87,88,89,90]
Sanitation	Chlorine-based compounds	Sprays	[79,80]
	Quaternary ammonium compounds	Sprays	[33,80,82,84]
Multi-technology	Pre-harvest		[48,54,62,65,124]
	Post-harvest		[108,109,111,115,116]

## 6. Conclusions and Future Directions

Adopting a comprehensive and integrative strategy for controlling *Salmonella* along the poultry production continuum allows for a robust framework that can be adopted for other pathogens and poultry species like turkey and duck production. These strategies will not only focus on eliminating existing pathogens but on preventing future contamination, thereby fostering a sustainable environment for poultry production. However, a potential limitation to the multi-hurdle multi-technology approach is the cost implication of adopting multiple strategies rather than a few single approaches. Therefore, research looking into risk assessments and cost benefits of this control measure is critical to the successful adoption of this multi-strategy approach. As the poultry industry evolves, the continual development and assessment of new interventions will be necessary to advance the control of foodborne pathogens and other pathogenic microbial populations effectively. The continuous evaluation and refinement of these interventions are essential to adapt to emerging challenges, such as evolving pathogen profiles and changing production practices. Studies focusing on innovative interventions and technologies intended to complement the established approaches will be crucial in mitigating microbial evolution. For example, research looking into the use of specific bacteriophages targeting specific *Salmonella* serotypes or a combination of bacteriophages targeting a wide range of serotypes is promising to eliminate serotypes of concern in poultry and reduce public health risks. This line of inquiry will represent efforts to utilize biological methods alongside traditional interventions, potentially offering additional pathways for pathogen control. Future advancements in technology and methods, including machine learning and microbiome studies, may further optimize the multi-hurdle approach, leading to improved control measures for *Salmonella* in poultry production.

## Data Availability

Not applicable.
